# The Influence of Host Aphids on the Performance of *Aphelinus asychis*

**DOI:** 10.3390/insects13090795

**Published:** 2022-08-31

**Authors:** Zhen-Xiang Li, Meng-Qi Ji, Chi Zhang, Yi-Bing Yang, Zhen-Zhen Chen, Hai-Peng Zhao, Yong-Yu Xu, Zhi-Wei Kang

**Affiliations:** 1 College of Plant Protection, Shandong Agricultural University, Tai’an 271018, China; 2 School of Life Science, Institutes of Life Science and Green Development, Hebei University, Baoding 071002, China; 3 Rural Energy and Environment Agency, Ministry of Agriculture and Rural Affairs, Beijing 100125, China; 4 Jinxiang County Agriculture and Rural Bureau, Jining 272200, China

**Keywords:** *Aphelinus asychis*, host aphids, biological performance, body size plasticity

## Abstract

**Simple Summary:**

Biological control is one of the most environmentally friendly and economically sound alternatives to chemical insecticides in many agricultural systems. How to improve the biological performance and mass-rearing methods of natural enemies has become the main factor restricting the application of biological control. *Aphelinus asychis* Walker is an important aphid parasitic wasp and has been successfully used to control several pest aphids in greenhouses and fields. In this study, we compared the biological performance and stress tolerance of *A. asychis*, which was reared on different aphids. The findings in this study will be useful for mass-rearing and releasing of *A. asychis* to control many pest aphids in greenhouses and fields.

**Abstract:**

The aphid parasitoid *Aphelinus asychis* Walker is an important biological control agent against many aphid species. In this study, we examined whether the rearing host aphid species (the pea aphid, *Acyrthosiphon pisum* and the grain aphid, *Sitobion avenae*) affect the performance of *A. asychis*. We found that *A. pisum*-reared *A. asychis* showed a significantly larger body size (body length and hind tibia length) and shorter developmental time than *S. avenae*-reared *A. asychis*. There was no difference in the sex ratio between them. The longevity of *A. pisum*-reared *A. asychis* was also significantly longer than that of *S. aveane*-reared *A. asychis*. Furthermore, *A. pisum*-reared *A. asychis* presented stronger parasitic capacity and starvation resistance than *S. aveane*-reared *A. asychi*. In addition, host aphid alteration experiments showed that *A. asychis* only takes two generations to adapt to its new host. Taken together, these results revealed that *A. pisum* is a better alternative host aphid for mass-rearing and releasing of *A. asychis*. The body size plasticity of *A. asychis* is also discussed.

## 1. Introduction

Aphids are among the most damaging agricultural pests of many economic plants due to their direct feeding and transmission of plant viruses [1,2]. In China, from 2006 to 2015, cereal aphids’ (the grain aphid, *Sitobion avenae* Fabricius, the greenbugs, *Schizaphis graminum* Rondani and the bird cherry-oat aphid, *Rhopalosiphum padi* Linnaeus) infestation in wheat caused around 930,000 tons of yield loss and USD 0.41 billion of economic loss every year [3]. Currently, management of pest aphids mainly relies on chemical insecticide applications [1,4]. However, due to the intensive use of insecticides, many aphid species have developed high resistance to various insecticides [4,5,6]. High insecticide resistance results in heavier insecticide application, leading to insecticide pollution and residues in the environment [7,8,9,10,11]. With increasing attention to the adverse effects of insecticides, a need for environmentally friendly management has arisen [7,10].

Parasitoids of aphids are one of the most important biological control agents, and some species are currently commercially available for aphid pest management [12]. However, many factors influence the fitness of parasitoids, thereby restricting their applications. Host quality is one of the most important factors that influence the fitness of parasitoid wasps [13,14,15,16]. “Adult size-fitness hypothesis” shows that body size is positively correlated with the fitness of hymenopteran parasitoids, suggesting that larger parasitoid wasps have more advantages, such as longer longevity and stronger competitiveness [17]. For example, *Drosophila hydei*-reared larger *Trichopria drosophilae* survived much longer than that reared on the smaller common fruit fly (*Drosophila* *melanogaster*-reared) in survival experiments [16]. Fitness evaluations of fly, whitefly and corn borers parasitoids on different hosts were conducted to figure out which host is more suitable for mass-rearing and releasing of these parasitoids [13,14,16,18]. In aphid parasitoids, *S. avenae* is one of the most common host aphids used for mass-rearing aphid parasitoids [19,20]. However, there is little information on the comparison between the fitness of the aphid parasitoids emerging from *S. avenae* and other aphid species.

*Aphelinus asychis* Walker (Hymenoptera: Aphelinidae) is an endoparasitoid of aphids that are spread worldwide. They can parasitize and prey on more than 40 aphid species [21]. In the United States, *A. asychis* has been successfully used to control the Russian wheat aphid *Diuraphis noxia* in the field [22,23]. It has been reported that 20 females of *A. asychis* can parasitize 4655 *S. graminum* during their entire lifespan [24]. Additionally, they feed by probing the aphid nymphs with the ovipositor, which also killed a total of 608 *S. graminum* [24]. Recently, we established a system of *A. asychis* using *S. avenae* as its host aphid to control *M. persicae* in chili pepper and cabbage [20]. In previous studies, *A. asychis* could successfully parasitize the pea aphid, *Acyrthosiphon pisum* (Harris) and finish its development [25,26]. However, whether *A. pisum* is a suitable host for mass-rearing *A. asychis,* or whether *A. asychis* reared on a larger host aphid (*A. pisum*) could be more advantageous for further biological control has never been examined.

The purpose of the current study was to compare the fitness-related traits of *A. asychis* reared on two different host aphids, *S. avenae* and *A. pisum*. Firstly, we determined the body size, developmental times, eclosion ratio, sex ratio and longevity of *A. asychis* reared on *S. avenae* (hereafter, SA-reared *A. asychis*) and *A. pisum* (hereafter, AP-reared *A. asychis*). Secondly, the parasitism of *A. asychis* on *S. avenae*, *A. pisum* and *M. persicae* was evaluated. Finally, we compared the starvation and thermal tolerance between *S. avenae* and *A. pisum*-reared *A. asychis*.

## 2. Materials and Methods

### 2.1. Insect Rearing

Adult *A. asychis* was originally collected from *S. avenae* on a winter wheat field in Shaanxi province, China. The laboratory colony of *A. asychis* was established using *S. avenae*, which is maintained on wheat (“Lumai 21”), and *A. pisum,* which is reared on broad beans (*Vicia faba* L., var. “Jingxuancandou”, Jinnong, Taigu, Shaanxi, China). The green peach aphid (*Myzus persicae)* colony was established on chili pepper (*Capsicum annuum* L., var. “Lingxiudajiao F1”). All of these insect colonies and plants were maintained in an air-conditioned insectary (Photoperiod: 16 h L:8 h D; Temperature: 25 ± 1 °C and humidity: 65 ± 5%).

### 2.2. Effects of Aphid Species on Some Life-History Traits of A. asychis

Two hundred of the second- or third-instar aphid (*S. avenae* or *A. pisum*) nymphs were placed on corresponding host plants and allowed to settle for 6 h. Then, the plant–aphid complex was transferred into a nylon mesh-covered plastic cage (Length × Width × Height: 19 × 16 × 28 cm). Five pairs (five females and five males) of two-day-old *A. asychis* were introduced into this plastic cage and allowed to parasitize for 24 h. Twenty-four hours later, the *A. asychis* were removed and all aphids were kept reared until mummification. The mummies were counted and separately transferred into 200 μL PCR tubes with small breathing holes. The mummies were kept under the rearing conditions described above and checked daily. The emergence date of the adult *A. asychis* was recorded. Males and females were discriminated and counted under a microscope. After they were frozen to death at −20 °C, the body length and hind tibia length of at least 30 *A. asychis* were measured under a microscope (Olympus SZX10 SZX2-ILLT).

The newly emerged *A. asychis* was introduced into the above-described plastic cage with one hundred second or third-instar aphids (*S. avenae* or *A. pisum*) and their corresponding host plants. The aphids and their corresponding host plants were changed daily. The longevity of thirty *A. asychis* females was checked daily from the first day of the experiment until their death.

### 2.3. Effects of Aphid Species on Parasitism of A. asychis on M. pericae

To determine the parasitic capacity of *A. asychis*, two pairs of two-day-old *A. asychis* were introduced into a plastic cage with two hundred of second or third-instar aphids (*S. avenae*, *A. pisum* or *M. persicae*) and their corresponding host plants, as described above. *A. asychis* was allowed to parasitize for 10 h and then was removed. The aphids were continuously maintained on their host plants until mummification. Mummies were counted and separately transferred into the above-described PCR tubes for adult emergence. The mummies were checked daily, and the number of emerged adults was recorded. Five biological replicates were conducted in this experiment.

### 2.4. The Starvation and Thermal Tolerance of A. asychis

Fifty newly emerged female adults of *A. asychis* (*S. avenae* and *A. pisum*) were separately transferred into a PCR tube, as described above. For starvation tolerance experiments, all of the PCR tubes containing *A. asychis* were kept under rearing conditions without access to aphid, water or plant. In this experiment, each female was treated as a biological replicate. For the thermal tolerance experiment, PCR tubes with *A. asychis* were placed into an electronic incubator at two high temperatures (37 and 39 °C) for 1 h, respectively [27,28]. One hour later, the number of surviving adults was recorded. Each treatment had three biological replicates, and the per biological replicate contained 30 female adults.

### 2.5. The Body Size Plasticity Analysis of A. asychis

To investigate the adaptation of *A. asychis* to a novel host, five pairs of two-day-old *A. asychis* emerging from *S. avenae* were introduced into a plastic cage as described above, with 200 second or third-instar nymphs of *A. pisum* and broad bean seedlings. Twenty-four hours later, these parasitoids were removed and the *A. pisum* was continuously reared until mummification. All mummies were collected and separated into 200 μL PCR tubes with small holes. Mummies were checked daily, and newly emerged adults were considered F1. The body length and hind tibia length of F1 adults were measured as described above. The number of days it took *A. asychis* to parasitize emerging adults was calculated as development time. Then, five pairs of two-day-old F1 *A. asychis* were introduced into an above-described plastic cage with 200 second or third-instar nymphs of *A. pisum* and broad bean seedlings. The experiment was conducted as described above, and F1’s offspring was F2. In order to obtain enough adults, 25 pairs of *A. asychis* and 1000 aphids per generation were used in this experiment. For each generation, we analyzed the body size and development time of at least 30 adults.

### 2.6. Statistical Analysis

In this study, all of these data were analyzed and visualized using GraphPad v9 (GraphPad Software, San Diego, CA, USA). The differences in body size (body length and hind tibia length), developmental time, sex ratio, parasitism, eclosion ratio, starvation tolerance and thermal tolerance between *A. pisum* and *S. avenae*-reared *A. asychis* were analyzed using Student’s *t*-test. The longevity of thirty *A. asychis* females was analyzed using the survival curve based on log-rank in GraphPad. Differences in body size (body length and hind tibia) among different generations in the body size plasticity experiment were determined through one-way analysis of variance (ANOVA) with separation of means via Fisher’s protected least significant difference (LSD) test at *p* < 0.05. As data on development time are not normally distributed, we used the nonparametric Kruskal–Wallis ANOVA test with Dunn’s multiple comparisons test to determine the differences between generations. All figures are presented with Mean ± SE.

## 3. Results

### 3.1. Effects of Aphid Species on Life-History Parameters of A. asychis

The body length and hind tibia length of both females (body length: *t* = 5.58, df = 58, *p* < 0.001; hind tibia length: *t* = 10.85, df = 58, *p* < 0.001; Table 1) and males (body length: *t* = 3.819, df = 70, *p* = 0.0003; hind tibia length: *t* = 5.120, df = 70, *p* < 0.001; Table 1) in AP-reared *A. asychis* were significantly longer than for SA-reared *A. asychis.* AP-reared *A. asychis* had shorter developmental time than the SA-reared *A. asychis* (*t* = 16.06, df = 75, *p* < 0.001; Table 1). There was no difference in the sex ratio between *A. asychis* released from *A. pisum* and *S. avenae* (*t* = 0.87, df = 8, *p* = 0.41; Table 1).

**Figure 1:**
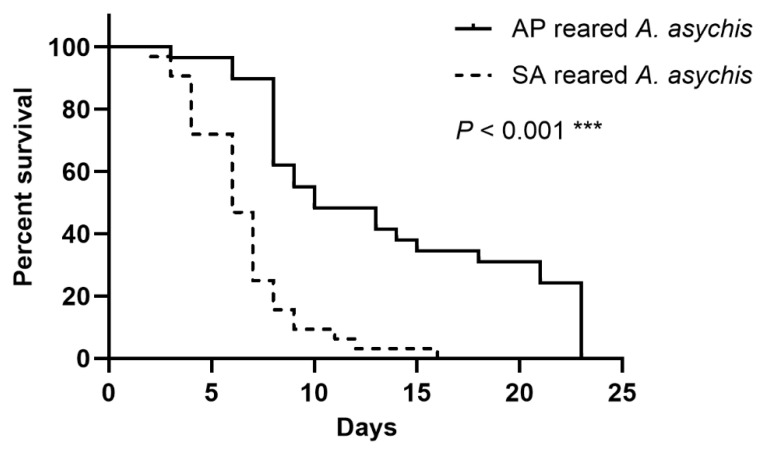
Percent survival of *A. asychis* emerging from *A. pisum* (AP-reared *A. asychis*) and *S. avenae* (SA-reared *A. asychis*) at 25 °C. *** means *p* < 0.001.

*A. asychis* females that emerged from *A. pisum* lived longer than those that emerged from *S. avenae* (χ^2^ = 27.47, df = 1, *p* < 0.001; Figure 1).

### 3.2. Effects of Aphid Species on Parasitic Capacity of A. asychis

**Figure 2:**
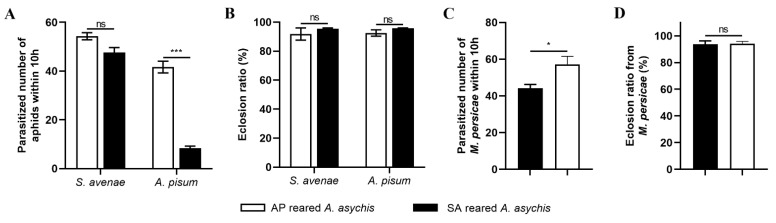
Parasitism of *A. asychis* emerging from *A. pisum* (AP-reared *A. asychis*) and *S. avenae* (SA-reared *A. asychis*). (**A**) Mean number of aphids parasitized within 10 h by *A. asychis* reared on nymphs of *A. pisum* or *S. avenae* (*n* = 3); (**B**) Eclosion rate of *A. asychis* reared on nymphs of *A. pisum* or *S. avenae* from *A. pisum* and *S. avenae* (*n* = 3); (**C**) Mean number of *M. persicae* parasitized within 10 h by *A. asychis* reared on nymphs of *A. pisum* or *S. avenae* (*n* = 5); (**D**) Eclosion rate of *A. asychis* reared on nymphs of *A. pisum* or *S. avenae* from *M. persicae* (*n* = 5). Mean ± SE, Student’s *t*-test. * means *p* < 0.05, *** means *p* < 0.001, and ns means no significant difference.

The parasitism capacity and eclosion ratio of AP-reared *A. asychis* and SA-reared *A. asychis* on the *S. avenae* aphid was similar (Parasitism: *t* = 2.673, df = 4, *p* = 0.0557; Eclosion: *t* = 0.8007, df = 4, *p* = 0.4682; Figure 2). When provided with *M. persicae* and *A. pisum*, AP-reared *A. asychis* parasitized more aphids than that of the SA-reared *A. asychis* (*A. pisum*: *t* = 1.319, df = 4, *p* = 0.2575; *M. persicae*: *t* = 2.706, df = 8, *p* = 0.0268). There was no difference in the eclosion ratio (*A. pisum*: *t* = 9.552, df = 4, *p* < 0.001; *M. persicae*: *t* = 0.1349, df = 8, *p* = 0.896; Figure 2).

### 3.3. Effects of Aphid Species on the Starvation and Thermal Tolerance of A. asychis

**Figure 3:**
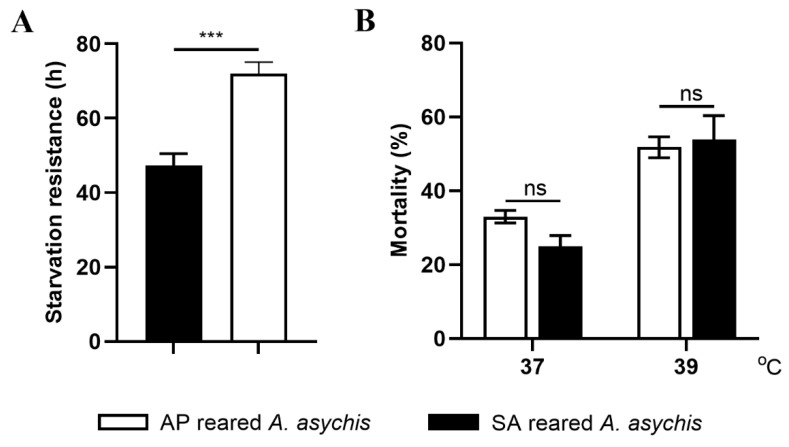
Starvation (**A**) and thermal tolerance (**B**) of *A. asychis* reared on nymphs of *A. pisum* (AP-reared *A. asychis*) or *S. avenae* (SA-reared *A. asychis*). Mean ± SE, Student’s *t*-test. Starvation: *n* ≥ 30; thermal tolerance: *n* = 3 (per replicates 30 female adults). *** means *p* < 0.001, and ns means no significant difference.

The starvation tolerance of AP-reared *A. asychis* was much stronger than that of SA-reared *A. asychis* (*t* = 5.552, df = 65, *p* < 0.001; Figure 3). The thermal tolerance of *A. asychis* released from *A. pisum* and *S. avenae* was similar at 37 and 39 °C (37 °C: *t* = 2.376, df = 4, *p* = 0.0763; 39 °C: *t* = 0.3104, df = 4, *p* = 0.7718; Figure 3).

### 3.4. The Body Size Plasticity of A. asychis

The body and hind tibia length of the F1 generation was longer than that of the F0 (Female body length: *F* = 11.82, df = 116, *p* = 0.0496; Female hind tibia length: *F* = 49.05, df = 116, *p* < 0.0001; Male body length: *F* = 5.253, df = 119, *p* = 0.0649; Male hind tibia length: *F* = 16.18, df = 119, *p* = 0.0314), and smaller than the >F10 generation (Female body length: *F* = 11.82, df = 116, *p* = 0.0072; Female hind tibia length: *F* = 49.05, df = 116, *p* = 0.0002; Male body length: df = 119, *F* = 5.253, *p* = 0.0312; Male hind tibia length: *F* = 16.18, df = 119, *p* < 0.0001;) (Table 2). Furthermore, the developmental times of F1, F2 and >F10 generations were significantly shorter than that of the F0 generation (F1: *H* = 77.98, df = 116, *p* < 0.0001; F2: *H* = 77.98, df = 116, *p* < 0.0001; >F10: *H* = 77.98, df = 116, *p* < 0.0001; Table 2).

## 4. Discussion

With the attention of biological control, numerous studies have been conducted to investigate how to improve the biological performance and mass-rearing methods of natural enemies [13,29,30,31]. Host insects have been identified as an important factor influencing the biological performance of natural enemies [15,18,32,33,34]. For example, the superworm, *Zophobas morio* pupae-reared *Dastarcus helophoroides* presented a better biological performance than that of the yellow mealworm beetle, *Tenebrio molitor* pupae-reared *D. helophoroides* [35]. Similarly, the body size of *Lysiphlebus testaceipes* and *L. fabarum* released from the fourth instar of aphid nymphs (*S. g* for *L. testaceipes* and the black bean aphid, *Aphis fabae* for *L. fabarum*) were significantly larger than those released from second-instar aphid nymphs [36,37]. Accordingly, since *A. pisum* is bigger than *S. avenae*, we found that *A. asychis* emerging from *A. pisum* were significantly larger than those emerging from *S. avenae*. Notably, when we transferred SA-reared *A. asychis* to *A. pisum*, we found that it only takes two generations to adapt to the new host aphids. In another *Aphidius* species, *A. ervi*, Henry et al. [38] observed that when provided with large, good-quality aphids (*A. pisum*), fitness increased after a single generation on the novel host. Meanwhile, when provided with low-quality aphids (foxglove aphids, *Aulacorthum solani* Kaltenbach), it took more than 40 generations [38]. All of these results indicated that host insects’ body size is correlated with the body size of parasitoids, and parasitoids such as *A. asychis* can adapt to a new host aphid rapidly, thereby improving their performance on new host aphids.

In insects, individuals with a larger body size usually have longer life spans, higher mating success rates and stronger parasitic capacity [16,32,33,39,40,41]. In this study, we found that the longevity of *A. pisum*-reared larger *A. asychis* was longer than that of *S. avenae*-reared smaller *A. asychis*. Furthermore, *A. pisum*-reared larger *A. asychis* also parasitized more *M. persicae* and *A. pisum* than *S. avenae*-reared smaller *A. asychis*, while the parasitic capacity of *A. asychis* on *S. avenae* was not affected by host aphids. In another aphid parasitoid, *L. testaceipes*, Vieira et al. [37] observed that the egg load was greater in larger females compared to that of smaller females. On the contrary, *B.* *tabaci*-reared smaller *E.* *formosa* parasitized more *B. tabaci* nymphs than *T.* *vaporariorum*-reared larger *E. formosa* [42]. This result is also in agreement with those of another two studies in *A. figuensis* and *E. formosa*, the parasitism rate of *A. gifuensis* and *E. formosa* on their natal hosts was significantly higher than on newly provided alternate hosts [43,44]. In addition, host plant is another factor that influences the fitness of aphids, and subsequently influences the parasitoid performance [45,46,47]. For example, the body size and development times of *A. gifuensis* emerging from *S. avenae* were significantly influenced by different wheat cultivars [19]. A parallel study also found that plant-mediated differences in the body size of *M. persicae* subsequently influenced the suitability of its parasitoid, *Aphelinus varipes* [47]. All of these results suggested that the longevity of parasitic wasps is positively correlated with their body size. Furthermore, the parasitic capacity was influenced not only by the body size, but also by their rearing experience.

In addition to the longevity and parasitic capacity, thermal tolerance and starvation resistance of insects are also correlated with their body size [16,28,29,48]. Previous studies showed that females of both *A. gifuensis* and *A. asychis* had a larger body size and were more tolerant to high temperatures than male *A. asychis* [16,28,29,49]. Chen et al. [16] found that the survival rates of *D. hydei*-reared larger *T. drosophilae* were higher than those of *D. melanogaster*-reared smaller *T. drosophilae* at higher temperatures (25 and 37 °C) and starvation treatments. However, in stressful conditions, it was suggested that the bigger size might be a disadvantage [50]. Ismail et al. [50] observed that after cold stress, the small females of *Aphidius ervi* produced more eggs at emergence than the large females. In this study, we found that the thermal tolerance of *A. asychis* was not influenced by host aphids, whereas the starvation tolerance was. In *Notonecta maculate*, a positive relationship was found between body size and starvation resistance [48]. In parasitoids, the adult body size is often correlated with its nutrient reservation [51,52,53]. Thus, we speculated that *A. asychis* individuals accumulated more nutrients, such as lipids, when developing in *A. pisum* than in *S. avenae*, thereby leading to stronger starvation tolerance. In this study, all of these results were generated from three sets of highly artificial constant temperature regimes. However, in the field, ambient temperatures fluctuate over time. Even daily temperature fluctuations can be very wide. Milosavljević et al. [54] and McCalla et al. [55] reported that temperature fluctuations significantly influenced the life-history parameters of *Diaphorencyrtus aligarhensis* and *Tamarixia radiata*, which are parasitoids of the Asian citrus psyllid, *Diaphorina citri* Kuwayama. Thus, there are still substantive uncertainties to be considered, such as the interaction of cyclic temperatures with the life-history parameters and thermal tolerance of *A. asychis*. Our future studies will focus on the impact of constant and fluctuating temperatures on the life-history parameters and thermal performance of *A. asychis*, and whether host aphids influence the performance of *A. asychis* at fluctuating temperatures.

In summary, our study found that *A. pisum*-reared larger *A. scychis* has many advantages, such as longer longevity, stronger parasitic capacity and greater starvation tolerance than *S. avenae*-reared smaller *A. asychis*. Thus, *A. pisum* can be a better alternative host aphid for mass-rearing and releasing *A. asychis* to control many pest aphids.

## Figures and Tables

**Table 1:** Body length, hind tibia length, developmental times and sex ratio of *A. asychis* that were reared on *A. pisum* and *S. avenae*.

Host	Body Length (mm)		Hind Tibia Length (mm)		Developmental Times (Days)	Sex Ratio
	Female	Male	Female	Male		
*A. pisum*	1.22 ± 0.02 a	0.93 ± 0.02 a	0.43 ± 0.01 a	0.36 ± 0.01 a	12.70 ± 0.14 b	0.56 ± 0.06 a
*S. avenae*	1.06 ± 0.02 b	0.83 ± 0.02 b	0.32 ± 0.01 b	0.32 ± 0.01 b	16.00 ± 0.15 a	0.62 ± 0.04 a

Different letters indicate significant difference between *S. avenae* and *A. pisum*-reared *A. asychis* (*p* < 0.05).

**Table 2:** Body size and developmental plasticity of *A. asychis* transferred from *S. avenae* to *A. pisum*.

Host Generation	Body Length (mm)		Hind Tibia Length (mm)		Developmental Time (Days)
	Female	Male	Female	Male	
F0 (Parental)	1.06 ± 0.02 c	0.83 ± 0.02 b	0.32 ± 0.01 c	0.32 ± 0.01 c	16.00 ± 0.15 a
F1	1.13 ± 0.02 b	0.82 ± 0.03 b	0.39 ± 0.01 b	0.28 ± 0.01 b	12.03 ± 0.19 c
F2	1.15 ± 0.02 ab	0.92 ± 0.03 ab	0.41 ± 0.1 ab	0.35 ± 0.01 a	12.77 ± 0.10 b
>F10 *	1.22 ± 0.02 a	0.93 ± 0.02 a	0.43 ± 0.01 a	0.36 ± 0.01 a	12.70 ± 0.14 b

* >F10 Means: *A. asychis* has been continuously reared on *A. pisum* over 10 generations. Different letters indicate significant differences among the different generations (*p* < 0.05).

## Data Availability

The data presented in this study are available in the article.
